# Overstating the evidence – double counting in meta-analysis and related problems

**DOI:** 10.1186/1471-2288-9-10

**Published:** 2009-02-13

**Authors:** Stephen J Senn

**Affiliations:** 1 Department of Statistics, University of Glasgow, Glasgow, UK

## Abstract

**Background:**

The problem of missing studies in meta-analysis has received much attention. Less attention has been paid to the more serious problem of double counting of evidence.

**Methods:**

Various problems in overstating the precision of results from meta-analyses are described and illustrated with examples, including papers from leading medical journals. These problems include, but are not limited to, simple double counting of the same studies, double counting of some aspects of the studies, inappropriate imputation of results, and assigning spurious precision to individual studies.

**Results:**

Some suggestions are made as to how the quality and reliability of meta-analysis can be improved. It is proposed that the key to quality in meta-analysis lies in the results being transparent and checkable.

**Conclusion:**

Existing quality check lists for meta-analysis do little to encourage an appropriate attitude to combining evidence and to statistical analysis. Journals and other relevant organisations should encourage authors to make data available and make methods explicit. They should also act promptly to withdraw meta-analyses when mistakes are found.

## Background

We live in an age of meta-analysis and would-be meta-analysts are constantly exhorted to find all the evidence. A popular tool for evaluating the quality of meta-analysis places great stress on the efforts that have been made to find all the relevant studies and the extent to which these efforts have been described [1,2]. Meta-analysts are advised to use funnel plots [3] or other similar devices in an attempt to establish if there has been any publication bias in favour of significant results and to calculate how many missing studies it would take to overturn their conclusions [4].

The reverse problem, however, of finding evidence that isn't there has received rather less attention, yet is surely just as, if not more, serious.

## Methods

In this article I describe various species of this problem, illustrating it with examples from leading medical journals, including *The Journal of the American Medical Association *(*JAMA*), *The British Medical Journal(BMJ), The Lancet *and *The New England Journal of Medicine *(*NEJM*). There is no attempt to quantify the extent of this problem except by remarking that it has not been particularly difficult to find the examples I have found. However, it is hoped that the examples will serve a useful purpose in putting would-be meta-analysts on their guard. Once the examples have been presented I shall offer some speculative remarks as to what factors might pre-dispose towards the problems exemplified and what might be done to improve the situation.

In choosing and presenting these examples, I should make one point clear. They are not being chosen to exemplify authorial incompetence. In fact many of the authors of the papers I discuss are rightly acknowledged as leading experts in the field of meta-analysis and most of the papers chosen are impressive in many respects. On the contrary, I shall argue in due course, that the problem is one that cannot be cured by *trust*. The cure is in *transparency*. As such, tools for evaluating the quality of meta-analyses are largely irrelevant. What is necessary is to make it easy to check the claims.

## Results

### Simple double counting of studies

A recent meta-analysis of the safety of anticholinergics in chronic obstructive pulmonary disease (COPD) by Singh et al [5] in *JAMA *affords an example. A problem with this meta-analysis are that studies were counted twice. For example, a publication by Brusasco et al was included [6]. However, this publication was itself a meta-analysis of two-studies [7] one of which, by Donohue et al [8], was also separately included by Singh et al. Thus the Donohue et al study was included twice, which is clearly inappropriate.

### Double counting of some aspects of studies

This error is slightly more subtle. Again *JAMA *affords an example. A meta-analysis by Kozyrskyj et al compared short and long course treatment of otitis media with antibiotics [9]. An unsatisfactory feature of this overview is that arms of the same study are counted more than once [10]. A number of the trials being summarised had more than two arms. The way that the authors chose to deal with this was to enter the control arm twice. Thus (say) treatment A was compared to C and then treatment B (say) was compared to C. The net effect was that C was counted twice.

For example, a trial by Hoberman et al [11] was included twice, apparently once with 375 patients and once with 386. However, the original data refer to two long courses of antibiotics in 178 and 189 patients respectively and to one short course with 197 patients. It appears that this short course has been counted twice by Kozyrskyj et al so that we have 178+197 = 375 and 189+197 = 386. This sort of double counting seems to have occurred on at least three occasions.

A similar case appears in the meta-analysis by Brocklebank [12] et al in the *BMJ *comparing metered dose inhalers (MDI) and other hand held devices for delivering corticosteroids in asthma. Figure 2 of that paper includes what appear to be two studies by Vidgren et al. In fact, there is only one study, a three armed cross-over trial[13] comparing Diskhaler^®^, Easyhaler^® ^and an MDI. Presumably, the data for the MDI have been included twice in the overall summary.

A slightly different form of a double counting of some information from a study occurs in the paper by Singh et al [5] already cited. Two studies by Casaburi [14,15] are included in the meta-analysis. However one was a preliminary report on short term results and the other is the full report at conclusion, including the short term data. Thus the short term data must have been counted twice.

### Accepting implausible claims for the precision of individual studies

A meta-analysis by Hackshaw et al [16] in the *BMJ *considered passive smoking. The method involved weighting reported log-odds ratios using reported (or calculated from confidence intervals) standard errors. However, Peter Lee, in an extremely important but sadly neglected article [17] in *Statistics in Medicine *has pointed out that the fact that the standard error for a log-odds ratio is approximately equal to the square root of the sum of the reciprocals of the frequencies in the corresponding four-fold table provides various lower bounds on the standard error. Conversely, a given standard error implies a minimum sample size. In fact for a given total sample size *N*, the split of cases and non-cases in exposed and unexposed groups that gives rise to the minimum standard error is an equal split of *N*/4 subjects in each cell. It follows, for example, that for any reported variance, *V *the total sample size, *N *must satisfy the requirement that *N *≥ 16/*V*. Similar inequalities exist for the total of any two cells and for the numbers in any given cell.

As Lee showed [17], at least one of the studies [18] included by Hackshaw et al [16] in their meta-analysis has impossibly low standard errors when examined in this way: the numbers of subjects are too few in view of the precision claimed.

### Imputing data

The meta-analysis by Brockelbank et al [12] already cited has ten within-arm within-study standard deviations equal to 100.0. There is no explanation of this fact and it appears that these standard deviations are imputed. In fact cross-over studies are being combined and it seems that the authors are forcing them into the parallel group framework that RevMan, the Cochrane Collaboration software required (at least in its earlier versions). In order to do this they have invented between-patient standard deviations that are, in fact, irrelevant to judging the outcome from a cross-over trial.

This is, in my view, a bad idea, although, it must be granted that this is a far less serious error than some others described, since, if anything, the evidence from the cross-over studies is likely to be understated since between-patient standard deviations are used. Nevertheless, it is an inappropriate approach that should be avoided.

However, not all attempts to impute data understate the evidence. For example, Nicholson et al [19], in a meta-analysis of depression as a prognostic factor in heart disease were able to identify 54 relevant studies. Unfortunately, six of these only recorded a lack of a significant association and did not give confidence intervals. Nicholson et al imputed an effect estimate of one to the studies and estimated the standard errors by regression on the number of patients.

This procedure cannot be endorsed. The value of unity chosen is the value that gives the least possible association but this overstates the lack of association. For example, a study by Hallstrom [20], that enrolled 795 women for 12 years follow up but for which only the result 'not significant' is available is *awarded *a relative risk (RR) of 1.0 with a confidence interval 0.6 to 1.7. However, the study by Ferketich [21] which is based on 5007 women followed for ten years has a *reported *RR of 1.0 with a wider confidence interval of 0.5 to 2.0. It is surely not appropriate to give a smaller study for which the relevant data have had to be guesstimated more weight than a larger one for which the data are available.

It would have been better in my opinion to have excluded the six studies with insufficient detail altogether.

### Spurious precision of individual trials

An interesting paper by Peters et al [22] considered Bayesian approaches to combining epidemiological observational data on humans with experimental data in animals and illustrated this using an investigation of trihalomethane exposure as a possible cause of low birthweight. They identified five epidemiological and eight toxicological studies in animals. However, in analysing the toxicological studies they treated the pups in litters of rats as independent observations rather than treating them as repeated measures on the dams. Since the number of pups, is of course, much higher than the number of dams this has the consequence of 'spurious precision' [23,24]. In other words, there is an overstating of the evidence.

### Inappropriate pooling of treatments

A very thorough and in many ways expert meta-analysis by Jüni et al in the *Lancet *looked at the risk of cardiovascular events under rofecoxib [25]. A number of different treatments, including placebo, naproxen and non-naproxen non-steroidal anti-inflammatory drug (NSAID) were considered as controls. Thus the meta-analysis compares rofecoxib to a mixture of controls. This is not, in itself illegitimate but one has to be quite clear about the purpose of such a meta-analysis. The relevant null hypothesis is 'rofecoxib is identical to all these comparators'. If and when this null hypothesis is rejected the alternative hypothesis that then follows is 'rofecoxib is different from at least one of these comparators'.

Jüni et al, were criticised by researchers at Merck, the makers of rofecoxib, for contravening a basic principle of meta-analysis, namely to pool like with like [26]. I disagree that there is such a principle. However, I also disagree with a conclusion that Jüni et al drew from their analysis.

They implied that their meta-analysis showed that rofecoxib was different to *each *comparator, including placebo, and indeed that this was already clear from data available by 2000. However to be able to assert *this *alternative hypothesis, it is necessary to have tested rofecoxib separately against each comparator and for such a meta-analysis the comparators cannot be pooled. In order to justify this claim, they carried out 'a test of interaction' for treatment effect by type of comparator (placebo, naproxen or non-naproxen NSAID) and used a non-significant result to justify pooling. (See, for example, table 2 of that paper.)

However, there are a number of problems with this procedure. The first is that the term *interaction *is misleading. It is actually *main effects *(for example the difference between naproxen and placebo) that it is necessary to prove are zero. This is important, since the situation is qualitatively different from a genuine test of interaction involving trials of different type, or patients of a different sort, as a stratum where the same treatment and control is being compared [27]. Under such circumstances it is a higher order effect (the interaction) that is assumed zero until proof to the contrary is available. Here it is an effect *that is of the same order *(placebo – naproxen) as the effect being examined (rofecoxib-naproxen) that is assumed to be zero.

Secondly, absence of evidence is not evidence of absence. Had Jüni et al wished to use the extremely large amount of information comparing rofecoxib to naproxen to produce a comparison to placebo they should have used the formal method of the putative placebo [28,29].

Thirdly, it  is clear that this procedure is easily abused. Given a great deal of data showing that treatment A (say) is better than control C (say), a small trial inadequately comparing treatment A to B would fail to show a significant 'interaction' and entitle one to pool B and A and use the combined data to prove that B was better than C.

I cannot leave this example, however, without pointing out that I do not believe that the fact that an advantage of naproxen to rofecoxib is not proof of a disadvantage of rofecoxib compared to placebo lets Merck, the developers of rofecoxib, off the hook. The gastric benefits of rofecoxib compared to naproxen were clearly shown in the same study [30] in 2000 that showed the cardiovascular benefits of naproxen to rofecoxib. From that point onwards patients should have been informed that one net benefit was being traded against another, whatever the explanation of either.

### Numerical slips

This is a sin to which I must plead guilty myself on occasion. Indeed, it is inherent to all scientific work that mistakes are made from time to time and are likely to be perpetuated. A beautifully described example comes in Primo Levi's essay 'Chromium' in *The Periodic Table *[31] in which, in a piece of chemical and statistical detection, he becomes suspicious of an unchallenged recipe that requires the addition of 'twenty-three drops of a certain reagent'. Eventually he finds an old file card bearing 'the direction to add "2 or 3" drops and not "23"'(p131).

In a discussion of Bayesian approaches to specifying prior distributions for random effect variances Lambert et al [32] used the data from Kozyrskij [9] to illustrate the problems with random effects analyses. I presented a frequentist alternative based on using proc nlmixed^® ^of SAS^® ^but what I did not realise at the time was that I had coded the main effects of the trials inappropriately. (It was my colleague Jim Weir who subsequently discovered my mistake.) Thus, where I claimed a point estimate of 0.39 with a standard error of 0.20, a corrected analysis gives 0.42 with a standard error of 0.19. The difference is small in this case but that is at least partly a matter of luck.

### Incomplete reporting

This is a rather different problem. There are a number of reported meta-analyses where it simply is almost impossible to check the authors' results with certainty. In particular where the following combination applies, that neither the method of statistical analysis is specified nor are the data from the original study fully available, then a great deal has to be taken on faith. The problem then becomes analogous to one of hearsay evidence in court. What is asserted may well be true but it is very difficult to call anybody to account to establish its reliability.

Consider, for example, a paper by Hrjobartson and Gotszche in the *NEJM *[33] which, considers the efficacy of placebos. This is an extremely interesting investigation that I have referred to elsewhere very positively [34] that points out that to establish the efficacy of placebo to the same degree of proof we require for standard treatments we need trials which have a control group for the placebo, that is to say no treatment. The authors perform a meta-analysis of all the three armed trials (treatment, placebo, no treatment) they can find. An appendix, available on the website gives results but neither it nor the main paper actually details the methods in sufficient detail for the results to be reproduced.

It might be thought that detailing the method is superfluous. In fact, however, there is a bewildering array of techniques possible for conducting a meta-analysis. In my paper *The Many Modes of Meta *[35] I identified three major data types: all studies used the same outcome and raw data are available, the same outcome but summary data only and different outcomes in different studies. I also identified at least nine different philosophical approaches that could be used to analyse summary measures. Many of these nine different approaches could be implemented in different ways. For example, in deciding to analyse binary data, one has to make a choice of risk scale: risk difference, relative risk, odds ratio. A much-cited paper by Newcombe [36] compares eleven different approaches to estimating confidence intervals for a risk difference for a single trial. In other words there are dozens of ways that binary meta-analyses alone could be performed.

## Discussion

What these examples show is that neither competence of the authors not prestige of the journal is any guarantee that the results of a meta-analysis do not need checking. Expert authors make mistakes that the review process does not correct. It therefore follows that an important standard by which a meta-analysis is to be judged is checkability. I propose that the following five points should be adopted by the community of meta-analysts and users if we are to improve the reliability of meta-analysis.

1. Be vigilant about double counting.

2. Make results checkable.

3. Describe approaches to analysis in detail.

4. Judge the meta-analysis not the analyst.

5. Create a culture of correction.

As regards the first of these, I hope that I have given sufficient examples to put potential users on guard. Although I consider that quality checklists, however good, are of little relevance when deciding whether to trust a meta-analysis, they are potentially useful in warning would-be analysts what to consider. In this respect, however, the current favourite, the Oxman and Guyatt score, is quite inadequate as it does not warn the user of potential problems. Furthermore it has a bias in favour of inclusion. The ten points included (see Oxman et al [2] page 1272), are

1. Were the search methods reported?

2. Was the search comprehensive?

3. Were the inclusion criteria reported?

4. Was selection bias avoided?

5. Were the validity criteria reported?

6. Was validity assessed appropriately?

7. Were the methods used to combine studies reported?

8. Were the findings combined appropriately?

9. Were the conclusions supported by the reported data?

10. What was the overall scientific quality of the overview?

Of the points, one, point 2, explicitly stresses the importance of being comprehensive and five (points 1,3,4,5 and 6) also address inclusion, whereas it would have to be a researcher who was already sensitised to the problem of double counting (say) who took point 8 as being a reminder to pay attention to this.

The implementation of my second proposal is partly constrained by resources. One inherent advantage that meta-analyses of the Cochrane Collaboration have over others is the amount of space that is allowed compared to journals. This is a point in their favour. However technological advances are making it easier for journals to match this through supplementary material provided on the web and this is what we have to strive for.

The third point requires a recognition and acceptance that meta-analysis is, contrary to what is sometimes maintained, not simple after all. It is not just a question of pushing data into some software sausage machine and waiting for a summary to appear. Empowering the statistically innocent to perform statistical analyses has its drawbacks. Many choices have to be made along the way and not all are uncontroversial. In consequence it is necessary to describe those choices in some detail.

The fourth point is that we should recognise that even experts can make mistakes and even those with motives we mistrust can have good arguments. There is a rather silly secondary literature of meta-analysis that seeks to award quality points for overviews from this or that source. Even if the quality instruments being used were appropriate (and they are not) the false positives and negatives in any screening procedure based on such class scores would be so numerous as to make the information nearly worthless in judging whether to trust an individual analysis. Consider the case of Lee's checks [17] and Hackshaw et al's meta-analysis [16]. Lee works as a consultant to the tobacco industry – enough reason to distrust him when passive smoking is being discussed, many would say. Hackshaw et al are public health experts with a considerable reputation. Enough grounds to trust them, many (including me) would claim. However, the trust or mistrust we have in the meta-analysts is irrelevant once we have got to the point of debating a scientific issue such as whether a quoted standard error must be too small.

My final point is that journals should devote more space to the correction of previous work and that we need a mechanism for flagging problems with papers once identified. For example, as far as I am aware, the *BMJ *has not issued notes correcting either of the two meta-analyses [12,37] mentioned in this article, despite the fact that the problems have been pointed out to the editors. Peter Lee [17] drew attention in *Statistics in Medicine *(*SiM*) to the problem with the *BMJ *paper on passive smoking but a recent paper [38] in *SiM *not only does not cite Lee but cites the paper on passive smoking and uses it to illustrate a method to deal with missing studies, the opposite of the *known *problem! The editors of the *Journal of The Royal Statistical Society Series C *refused to publish a letter by Andy Grieve and me pointing to some problems with Peters et al [22], including that mentioned here. Over *two years *after I informed the Cochrane Collaboration regarding the double counting in the otitis media meta-analysis [39], there is still no correction. The editors of *JAMA *initially declined to take any action regarding the corresponding paper [9] when I brought it to their attention and I still wait to see what they will do about it.

## Conclusion

What is needed is an awareness that scientific progress occurs through an ongoing, vigorous process of debate and criticism and not through the piling up of incontrovertible facts. We must be prepared to check and correct (if necessary) published results and they must be published in a way that makes this easy.

## Competing interests

I act regularly as a consultant to the pharmaceutical industry. Companies I have advised include Boehringer Ingelheim, the manufacturers of tiotropium, which is mentioned in one of the meta-analyses I discuss. Since I am an academic, my career is furthered by publishing.

## Pre-publication history

The pre-publication history for this paper can be accessed here:

http://www.biomedcentral.com/1471-2288/9/10/prepub
